# Clinical and Radiologic Features of Fulminant Pediatric Autoimmune Encephalitis: A Case Report

**DOI:** 10.21980/J8JW75

**Published:** 2022-04-15

**Authors:** Raymen Rammy Assaf

**Affiliations:** * Harbor UCLA Medical Center, Department of Pediatric Emergency Medicine, Torrance, CA; ^ Children’s Hospital of Orange County, Emergency Medicine Specialists of Orange County, Orange, CA

## Abstract

There is a growing body of literature and scientific discovery on pediatric autoimmune encephalitides. This is a heterogenous group of neuroinflammatory conditions leading to often perplexing acute and progressive neurologic and/or neuropsychiatric symptoms. This report demonstrates the evaluation and management of an adolescent with myelin-oligodendrocyte glycoprotein (MOG) antibody-associated fulminant acute disseminated encephalomyelitis (ADEM) in the context of current literature. The featured magnetic resonance imaging uniquely highlights progressive central nervous system lesions detected over the course of the first week of disease.

**Topics:**

Encephalitis, encephalopathy, ADEM, MOG.

**Figure:**
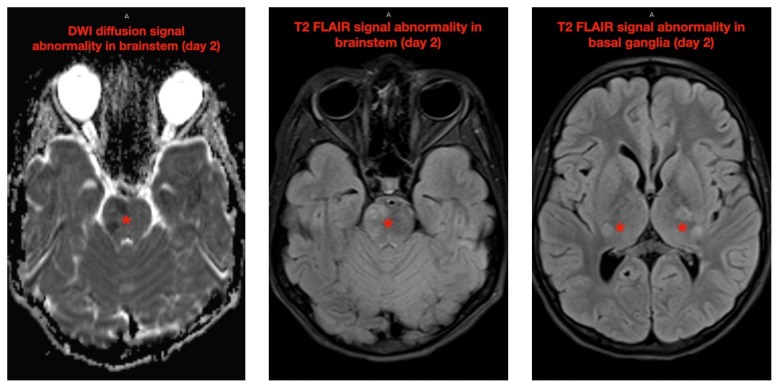


**Figure:**
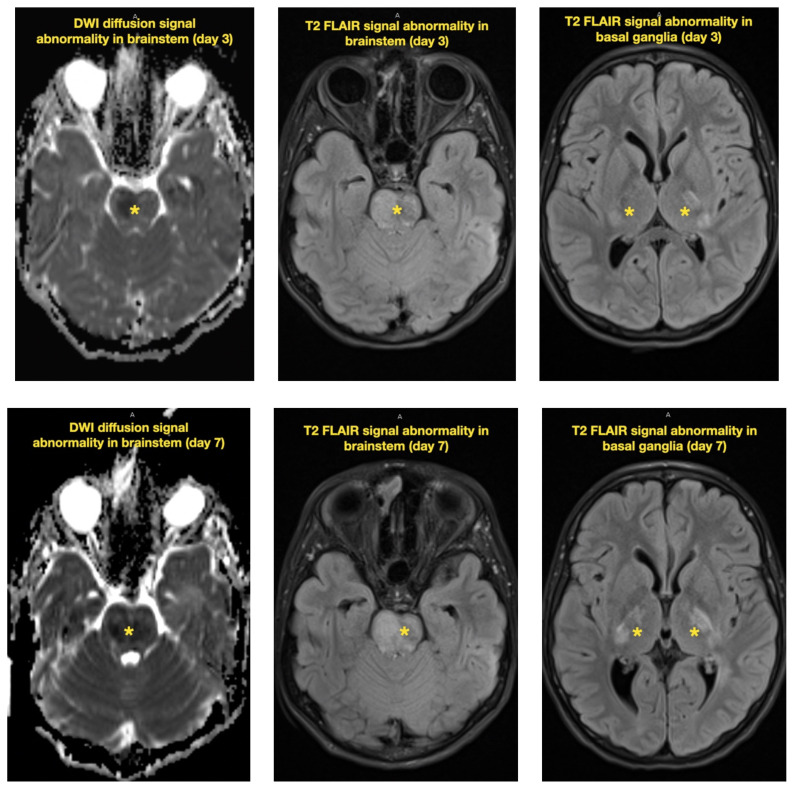


**Figure:**
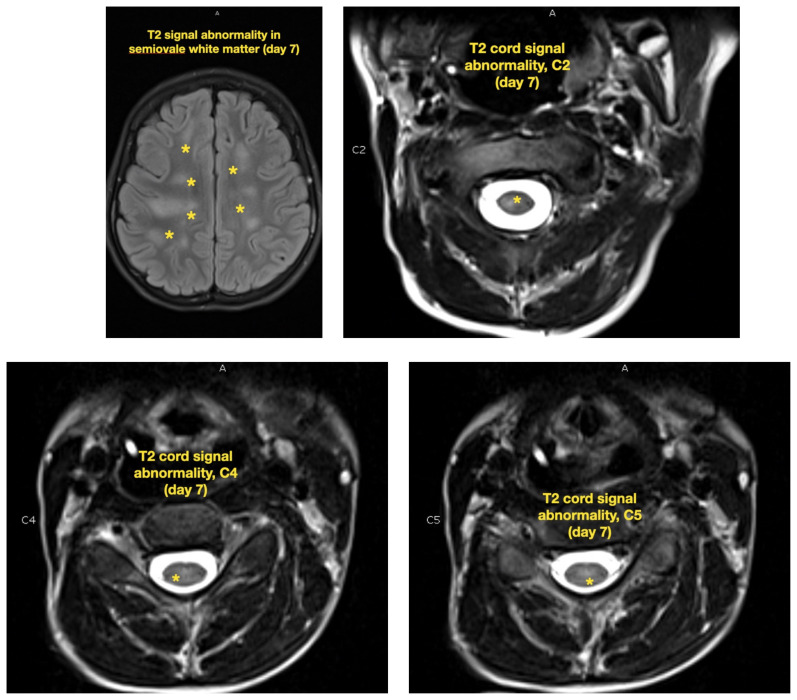


**Figure:**
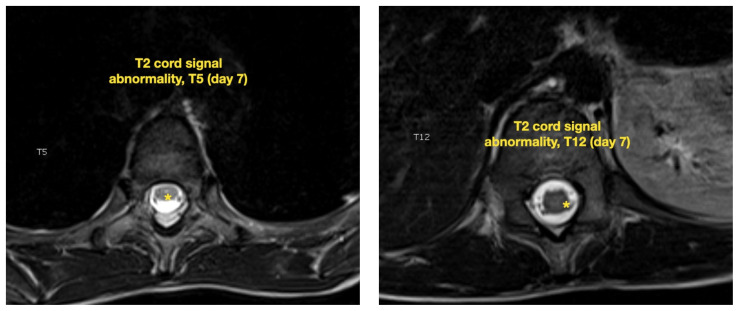


## Brief introduction

Autoimmune neuroinflammatory disorders occur in both children and adults and are characterized by antibodies directed against neuronal cell surface and myelin proteins, ion channels or specific receptors.^1^,^2^ Symptoms and signs are acute to subacute, dynamic, and often progressive, including behavior change, psychosis, headache, cognitive dysfunction, altered mental status, abnormal movements, dysautonomia, and seizures.^1^ Since symptoms are broad and often require a prolonged period for confirmatory testing, a high index of suspicion is required to avoid or minimize diagnostic and/or therapeutic delay. This case report demonstrates the management of an adolescent with myelin-oligodendrocyte glycoprotein (MOG) antibody-associated fulminant acute disseminated encephalomyelitis (ADEM).

## Presenting concerns and clinical findings

A healthy, fully vaccinated, 11-year-old boy presented with one day of progressive headache, non-bilious emesis and worsening confusion with generalized weakness. There was no associated syncope, seizure, preceding trauma or difficulty breathing. He had no recent fever, weight loss, night sweats, neck stiffness or cough. There was no recent travel and no exposure to medications or toxic/recreational substances. Prior to the onset of his symptoms, the patient was described by parents as a healthy and happy boy active in soccer. There was no family history of autoimmune disease.

On exam, the patient was afebrile, tachycardic (heart rate 110 beats/min), normotensive, slightly tachypneic (24 breaths/min), and with normal oxygen saturation. He had a Glasgow Coma Scale of 14, with spontaneous eye opening, and confusion (mumbling name softly with no response to other questions) but able to follow simple commands. He was normocephalic with supple neck and no nuchal rigidity; extraocular movements intact and pupils equal, round, and reactive to light; non-labored respirations with clear lungs to auscultation; soft abdomen with no palpable masses; and no rash or limb swelling, atrophy or contracture. Neurologically, the patient was lethargic (reduced wakefulness and disinterest in environment) with left-sided facial droop but without other cranial nerve deficits. He had normal sensation to light touch throughout; reduced left-sided strength with left upper extremity 2/5 and left lower extremity 3/5 strength; normal finger-to-nose; normal brachial, patellar, and Achilles tendon reflexes; gait was unable to be assessed due to patient’s clinical state.

A stat head CT obtained in the emergency department (ED) did not demonstrate mass, midline shift, intracranial hemorrhage or hydrocephalus. Laboratory evaluation showed a normal complete blood cell count with differential, complete metabolic panel, magnesium, phosphorus, erythrocyte sedimentation rate (ESR) and C-reactive protein (CRP), and lactate dehydrogenase level. Ammonia level, ethanol levels, and urine toxicology were normal. A venous blood gas was unrevealing, with no acidosis or lactate elevation. TSH and free T4 levels were both mildly depressed, suggesting a sick euthyroid state. Comprehensive respiratory viral panel testing by PCR was negative, including COVID-19. Lumbar puncture was unremarkable, with no pleocytosis or derangement in protein or glucose levels and a negative PCR meningitis panel (including HSV-1 and HSV-2).

## Significant findings

The neurology service was consulted in the ED and multisequence MRI and MR angiography (MRA) of the brain were obtained without and with IV contrast. Diffusion-weighted imaging (DWI) and T2-weighted-Fluid-Attentuated Inversion Recovery (FLAIR) sequences showed multifocal small areas of diffusion signal abnormality in the brainstem and basal ganglia (red asterisks) suggestive of ischemia. Additional multifocal bilateral supra- and infratentorial foci of signal abnormality including subcortical white matter and deep grey matter were highly concerning for encephalitis or demyelinating disease. MRI was repeated on day 3 and day 7 during evolution of disease.

## Patient course

The patient was admitted to the pediatric intensive care unit (PICU) for close monitoring and therapeutic intervention. His neurologic status deteriorated over the following 48 hours, becoming completely obtunded with decorticate posturing and Glasgow Coma Scale (GCS) waxing and waning between 5 to 9. Despite this, he continued to handle secretions well with no airway compromise on exam and normal carbon dioxide end tidal monitoring. Subsequent MRI imaging of the brain and entire spine obtained on day 3 and day 7 of disease demonstrated progression of lesions, including increasingly prominent and confluent multifocal areas of white matter, basal ganglia, and brainstem restricted diffusion (yellow arrows). Spinal imaging showed patchy multifocal asymmetric cord signal involving the right C2, central anterior C5, and central cord at T4, T5, and T12 (yellow asterisks).

An extensive infectious workup was unrevealing. This included blood, urine and cerebrospinal fluid (CSF) culture; Ebstein Barr Virus serology (blood and CSF); West Nile Virus serology (blood and CSF), Rickettsia serology, St. Louis Encephalitis serology, tuberculosis QuantiFERON testing, and Bartonella henselae and Quintana serology. *Mycoplasma pneumoniae* IgM blood antibody returned positive while IgG was equivocal. A similarly expansive rheumatologic workup was negative, including anti-nuclear antibody (ANA), complement levels and rheumatoid factor, as well as dsDNA, Smith IgG, SS-A, SS-B, Scl-70, and ribosomal P protein auto-antibodies. Neurologic lab work showed no oligoclonal bands or paraneoplastic antibodies in the CSF; however, serum myelin-oligodendrocyte glycoprotein (MOG) antibody – which is associated with ADEM - was strongly positive.

The patient was empirically treated for presumed autoimmune encephalitis with methylprednisolone and intravenous immune globulin (IVIG) followed by 7 days of plasmapheresis (PLEX). The patient’s neurologic status steadily improved during therapy, communicating using eye movements and intentional vocalizations, as well as improved truncal and neck strength. He subsequently initiated rituximab therapy once every 2 weeks with progressive improvement in functional neurologic status over the course of several weeks of intensive physical and occupational therapy. Five months following his original presentation, the patient has normal speech, ambulates with assistance of a walker and has residual left sided weakness, and is receiving ongoing physical and occupational outpatient therapy.

The patient’s clinical presentation and course along with repeated MR imaging was most consistent with fulminant MOG antibody-associated ADEM. Because this heterogenous disease most often occurs in the post-infectious phase, and an extensive infectious evaluation was performed, it was suspected that *Mycoplasma pneumoniae* may had been the pathologic trigger in this patient’s case.

## Discussion

There are a reported 7.3 cases of encephalitis per 100,000 person years in US children, peaking in infants (13.5 per 100,000).^3^ There has been an increase in incidence over the last decade which is attributed to increased sensitivity of MRI brain parenchymal imaging and the increased use of immunosuppressive therapies and organ transplantation.^3^ The International Encephalitis Consortium (IEC) criteria for clinical diagnosis is altered mental status longer than 24 hours without an alternative cause, with at least 3 supplemental criteria met for “probable” or “confirmed” diagnosis: fever ≥38oC within 72 hours, seizures, new focal neurologic findings, CSF pleocytosis, brain parenchymal changes on neuroimaging, or suspicious EEG findings.^3^

The most prominent cause of pediatric encephalitis is infection – most commonly herpes simplex virus (HSV) in developed Western countries – and worldwide, Japanese encephalitis is the most common neurotropic viral agent.^4^ The California Encephalitis Project found that autoimmune encephalitis (AIE) was collectively more common than any single infectious agent alone.^5^ In this group of neuroinflammatory disease, autoantibodies bind to neuronal membrane antigens or synaptic receptors, causing an often reversible neuronal dysfunction.^6^ Anti-N-methyl-D-aspartate receptor encephalitis (NMDAR-E) and ADEM have been described as the most frequent causes of AIE, with other neuronal antibodies only sporadically described in pediatric literature.^7^,^8^ CNS autoimmunity may be triggered by non-neuroinvasive pathogens (eg, *Mycoplasma pneumoniae*, enterovirus, influenza), neuroinvasive pathogens (eg, HSV), paraneoplastic syndrome (eg, ovarian teratoma, neuroblastoma), and possibly some vaccinations.^3^ Symptoms are well characterized and include an often dynamic combination of both psychiatric (psychosis, compulsive behavior, aggression, memory loss, fear or euphoria) and neurologic manifestations (abnormal movement, weakness, cognitive decline, decreased level of consciousness, and seizures), with the former often appearing earlier in the disease.^9^,^10^

Given some overlap in acute presentations, the first diagnostic step is to assess for possible ischemic or hemorrhagic stroke with CT head imaging. The diagnostic cornerstones of encephalitis, however, are 1) lumbar puncture with opening pressure, cell count with differential, glucose, protein, and diagnostic testing including oligoclonal bands (often repeated for persistent or worsening symptoms), 2) brain and spine MRI with and without contrast using diffusion, T2-weighted, and FLAIR sequences for brain parenchymal inflammation, and 3) EEG for encephalopathy, localizing signs, or subclinical seizure.^3^,^8^,^10^ Brain MRI is abnormal in up to 70% of patients with AIE, and patients with ADEM exhibit large, hazy, poorly demarcated asymmetrical white matter lesions in the corpus callosum, brainstem, cerebellum, and spinal cord, and deep grey matter involvement frequently in the thalamus.^11^,^12^ Patients with NMDAR-E often have a normal brain MRI.^11^ Anti-MOG antibody seropositivity during a first acute demyelinating syndrome has recently been described in multinational studies to occur in about 40 percent of all pediatric acute demyelinating syndrome presentations and is associated with ADEM, optic neuritis, and transverse myelitis.^12^ Notably, patients with MOG antibody-associated ADEM are at higher risk of developing epilepsy after acute inflammation.^12^ Even with complete diagnostic workup, a clear etiology may not be found in about 20 to 50 percent of children, making it important to consider a broad differential diagnosis.^3^,^8^

The therapeutic approach to encephalitis is primarily supportive, with close attention to the airway in cases of severely depressed mental status and/or bulbar dysfunction, targeted fluid and electrolyte goals given risk of SIADH (syndrome of inappropriate antidiuretic hormone), monitoring for development of cerebral edema, seizure control, and early initiation of rehabilitory therapy.^3^ Patients are typically placed on acyclovir while HSV diagnostic evaluation is pending.^13^ After a thorough assessment for active infectious etiology, an immunosuppressive regimen is implemented often before definite antibody diagnosis.^8^,^14^ This consists of corticosteroids, IVIG, and plasma exchange (PLEX), with second line therapies primarily rituximab and cyclophosphamide.^3^,^15^,^16^

This case illustrates acute onset encephalitis in an adolescent found to have MOG antibody-associated ADEM. The emergency physician should consider autoimmune encephalitis in any child with unexplained acute or subacute encephalopathy, with NMDA-R and ADEM composing the majority of these cases. A thorough infectious workup along with lumbar puncture, MRI brain/spine, and EEG are paramount and minimize diagnostic delay.

## Supplementary Information


















