# Supplementary figures and images for: Clinical and Radiologic Features of Fulminant Pediatric Autoimmune Encephalitis: A Case Report
Source: J Educ Teach Emerg Med. 2022 Apr 15;7(2):V21–7. doi: 10.21980/J8JW75 (PMC10332748; doi:10.21980/J8JW75)

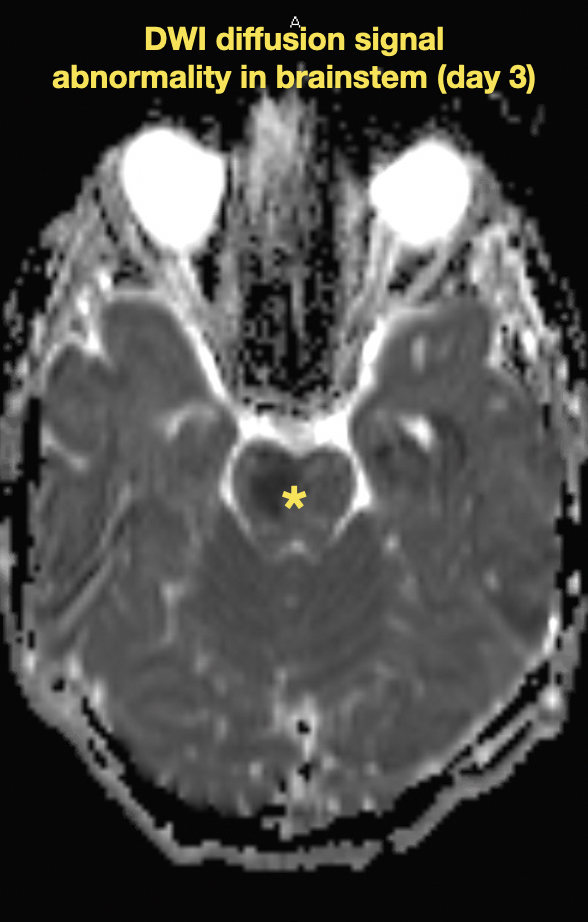

Supplement: Supplementary file 1 [file JETem-7-2-V21-supp1.jpg]

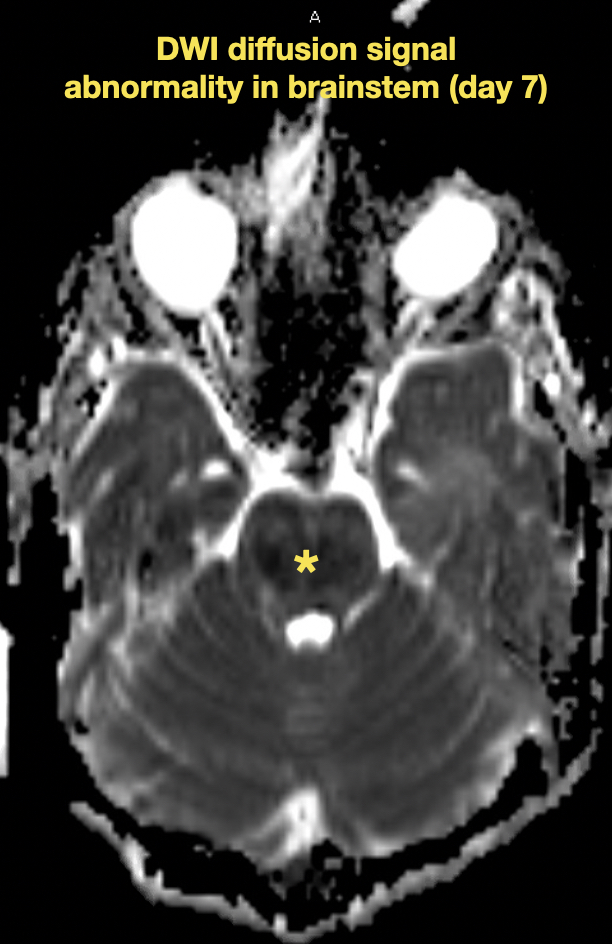

Supplement: Supplementary file 2 [file JETem-7-2-V21-supp2.jpg]

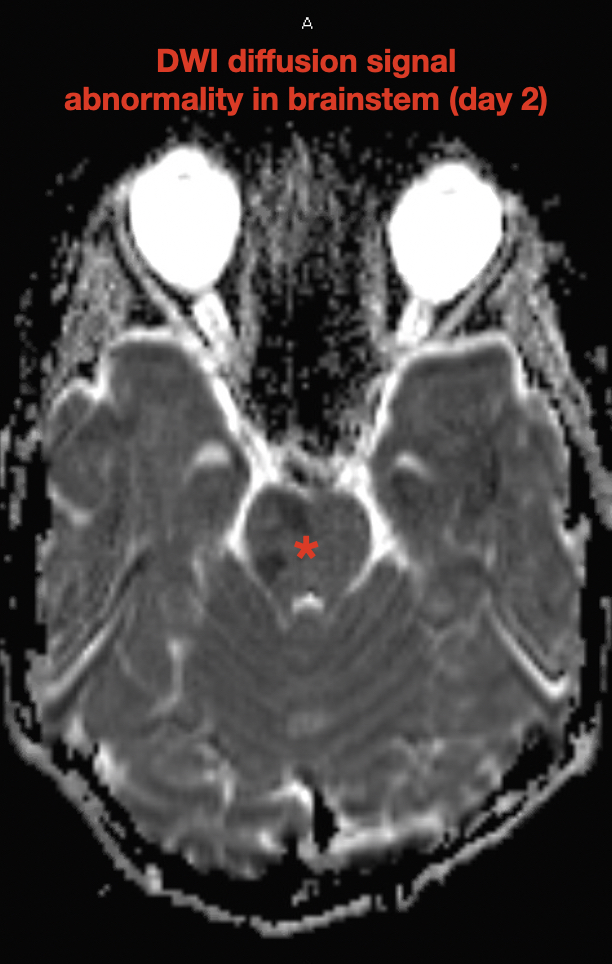

Supplement: Supplementary file 3 [file JETem-7-2-V21-supp3.jpg]

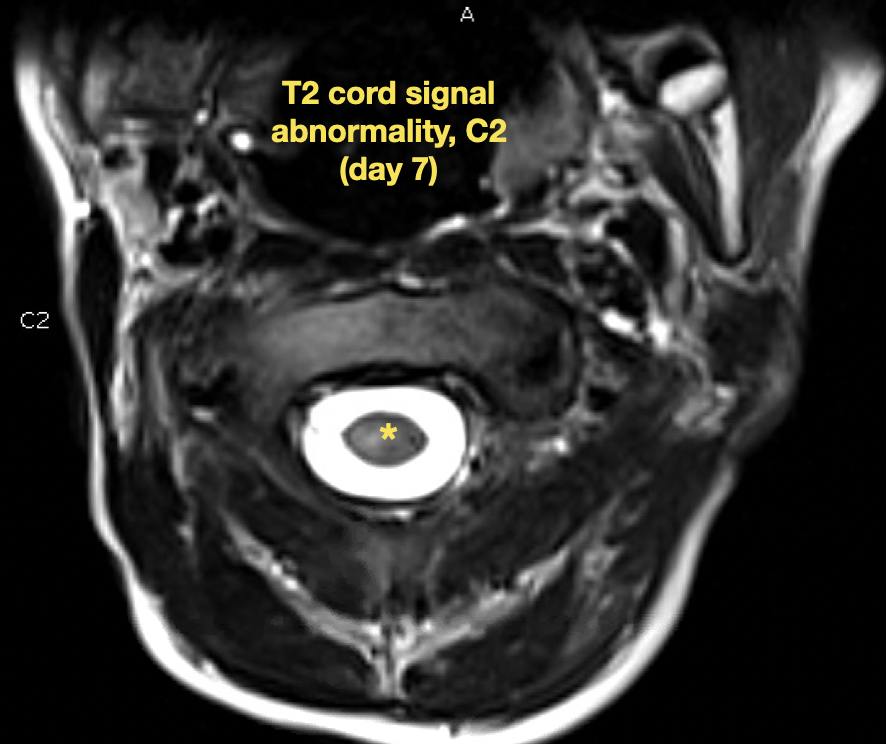

Supplement: Supplementary file 4 [file JETem-7-2-V21-supp4.jpg]

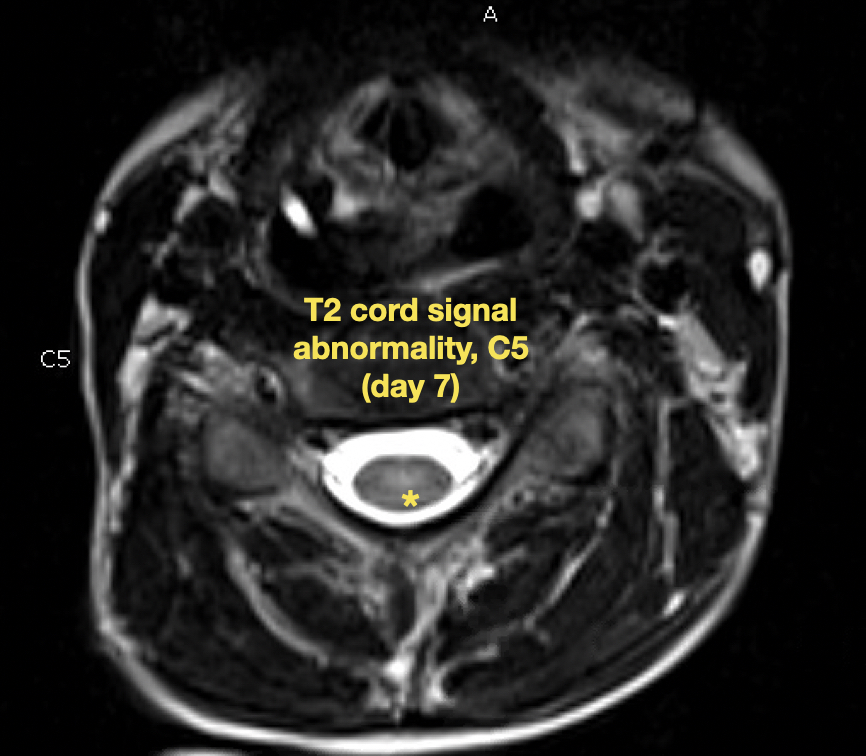

Supplement: Supplementary file 5 [file JETem-7-2-V21-supp5.jpg]

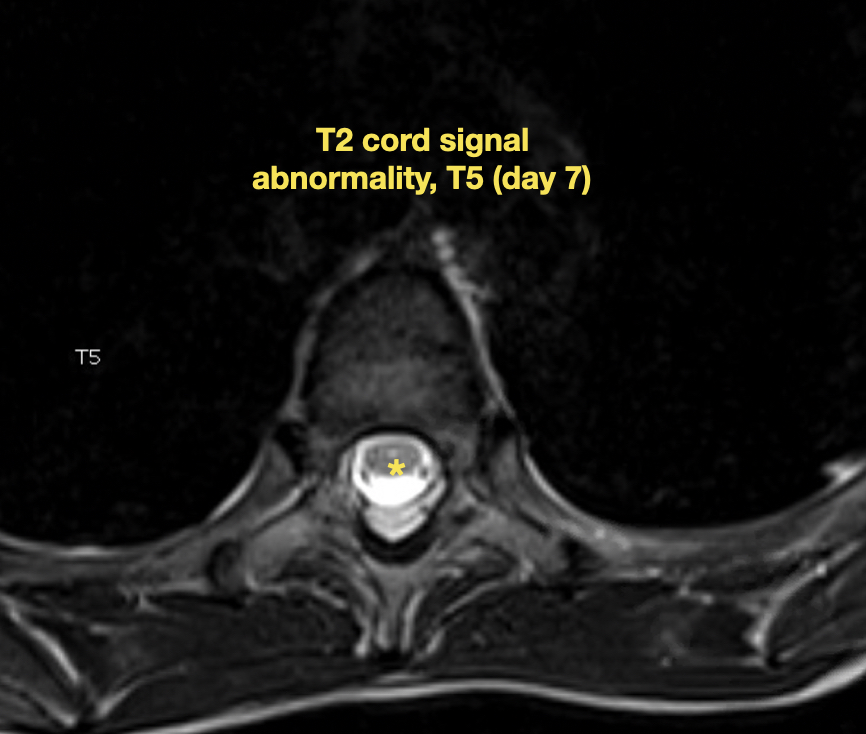

Supplement: Supplementary file 6 [file JETem-7-2-V21-supp6.jpg]

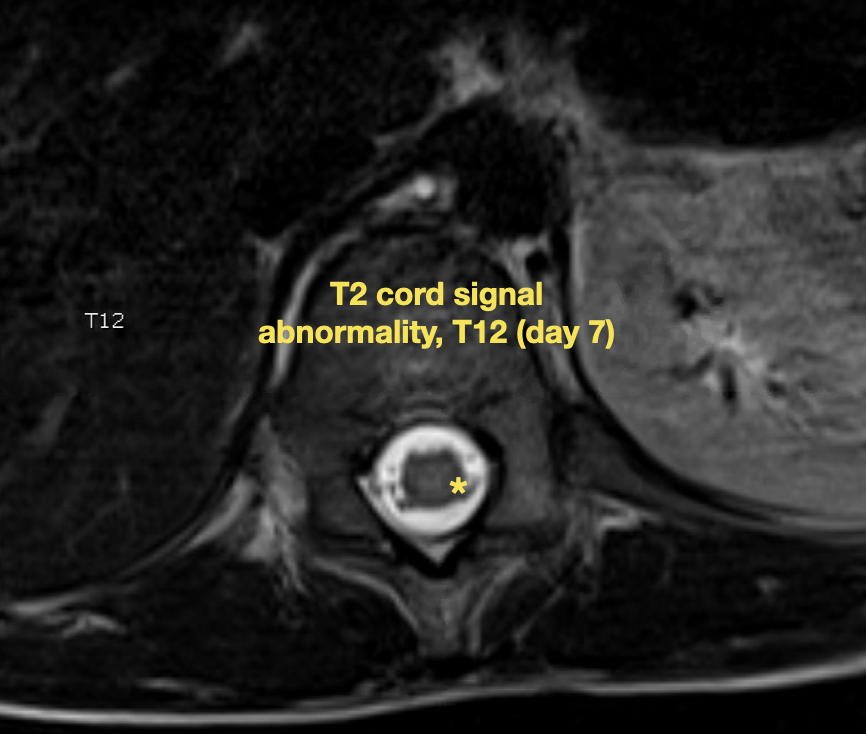

Supplement: Supplementary file 7 [file JETem-7-2-V21-supp7.jpg]

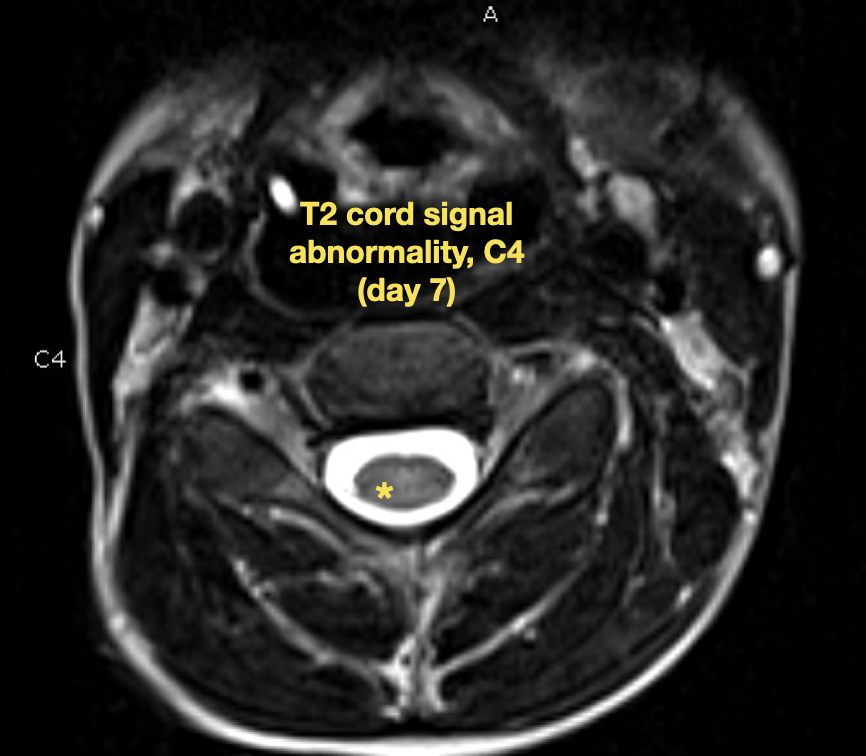

Supplement: Supplementary file 8 [file JETem-7-2-V21-supp8.jpg]

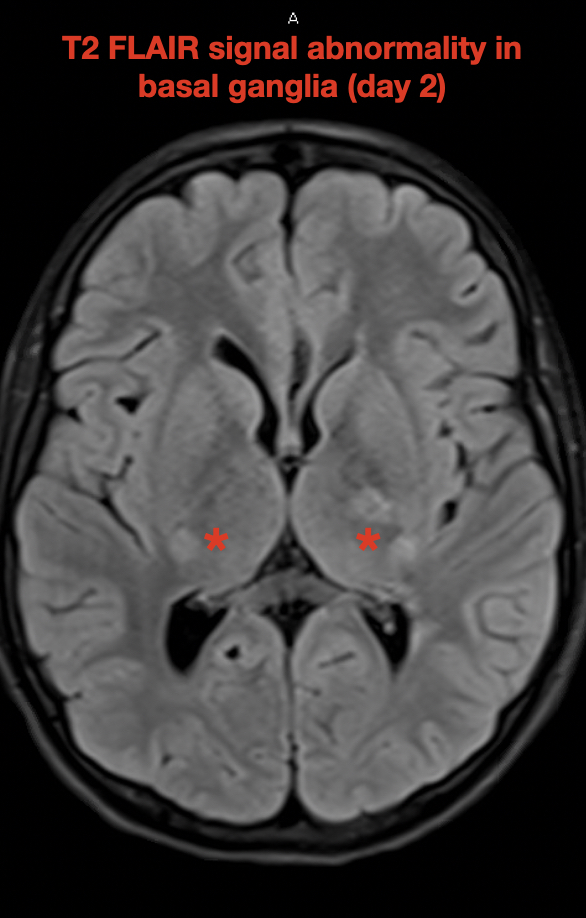

Supplement: Supplementary file 9 [file JETem-7-2-V21-supp9.jpg]
